# A comparison between a white LED confocal imaging system and a conventional flash fundus camera using chromaticity analysis

**DOI:** 10.1186/s12886-019-1241-8

**Published:** 2019-11-19

**Authors:** Valentina Sarao, Daniele Veritti, Enrico Borrelli, Srini Vas R. Sadda, Enea Poletti, Paolo Lanzetta

**Affiliations:** 1 Department of Medicine – Ophthalmology, University of Udine, Piazzale Santa Maria della Misericordia, 33100 Udine, Italy; 2 Istituto Europeo di Microchirurgia Oculare – IEMO, 33100 Udine, Italy; 3 Department of Medicine and Science of Ageing, Ophthalmology Clinic, University G. d’Annunzio Chieti-Pescara, Chieti, Italy; 4 Doheny Eye Institute, Doheny Image Reading Center, California, Los Angeles USA; 5 Department of Ophthalmology, David Geffen School of Medicine at UCLA, University of California, California, Los Angeles USA; 6 CenterVue SpA, Padua, Italy

**Keywords:** Chromaticity, Confocal white LED system, Conventional flash fundus camera, Eidon, Topcon

## Abstract

**Background:**

Conventional flash fundus cameras capture color images that are oversaturated in the red channel and washed out in the green and blue channels, resulting in a retinal picture that often looks flat and reddish. A white LED confocal device was recently introduced to provide a high-quality retinal image with enhanced color fidelity. In this study, we aimed to evaluate the color rendering properties of the white LED confocal system and compare them to those of a conventional flash fundus camera through chromaticity analysis.

**Methods:**

A white LED confocal device (Eidon, Centervue, Padova, Italy) and a traditional flash fundus camera (TRC-NW8, Topcon Corporation, Tokyo, Japan) were used to capture fundus images. Color images were evaluated with respect to chromaticity. Analysis was performed according to the image color signature. The color signature of an image was defined as the distribution of its pixels in the *rgb* chromaticity space. The descriptors used for the analysis are the average and variability of the barycenter positions, the average of the variability and the number of unique colors (NUC) of all signatures.

**Results:**

Two hundred thirty-three color photographs were acquired with each retinal camera. The images acquired by the confocal white LED device demonstrated an average barycenter position (*rgb* = [0.448, 0.328, 0.224]) closer to the center of the chromaticity space, while the conventional fundus camera provides images with a clear shift toward red at the expense of the blue and green channels (*rgb* = [0.574, 0.278, 0.148] (*p* < 0.001). The variability of the barycenter positions was higher in the white LED confocal system than in the conventional fundus camera. The average variability of the distributions was higher (0.003 ± 0.007, *p* < 0.001) in the Eidon images compared to the Topcon camera, indicating a greater richness of color. The NUC percentage was higher for the white LED confocal device than for the conventional flash fundus camera (0.071% versus 0.025%, *p* < 0.001).

**Conclusions:**

Eidon provides more-balanced color images, with a wider richness of color content, compared to a conventional flash fundus camera. The overall higher chromaticity of Eidon may provide benefits in terms of discriminative power and diagnostic accuracy.

## Background

Color fundus photography is an important tool in the diagnosis and monitoring of various retinal diseases. Clear and detailed photographs allow for an accurate evaluation of the ocular fundus and provide a precise documentation of retinal findings that can be archived, shared for telemedicine applications, or used as a valuable educational tool [1]. In digital retina cameras, a bright flash is used to illuminate the ocular fundus; the light reflected is then captured on the pixel array of a charge-coupled device, and a digital image is subsequently generated. Conventional fundus cameras illuminate large areas of the retina, typically with a flash lamp, and capture 35–45 degree, high-resolution digital images. Currently, color images acquired with traditional fundus cameras continue to play a pivotal role in the documentation, diagnosis and monitoring of retinal disorders [2, 3]. However, conventional flash devices frequently capture color images that are oversaturated in the red channel and washed out in the green and blue channels, yielding a retinal picture that often looks flat and reddish [3].

There is increasing recognition that the use of specific wavelengths and confocal technologies can help with better detection and delineation of fundus images [4–6].

Confocal systems allow the capture of reflected light through a small confocal aperture, which suppresses any scattered or reflected light outside the focal plane that could potentially blur the image. This results in a sharp, high-contrast image of an object layer located within the focal plane [7].

In contrast, traditional fundus cameras use a flash lamp with a broad spectrum illumination; in the absence of confocal optics, the reflected signal is derived from all tissue levels in the beam of the flash of light, and light scattering anterior and posterior to the plane of interest can greatly influence the detected signal [8].

For that reason, more recent fundus imaging systems have taken advantage of principles of confocal technology.

The advantages of using confocal optics over traditional flash-based systems include improved image quality, enhanced contrast, more finely detailed images, suppression of scattered light, and better imaging of patients with poor dilation [9, 10]. Recently, a nonmydriatic system that combines confocal technology with white light emitting diode (LED) illumination, has been introduced to provide a high quality retinal image with enhanced color fidelity (Eidon, Centervue, Padova, Italy) [11, 12].

Color images acquired by traditional fundus camera and by this new, white LED confocal device, however, differ in color rendering, and the differences in chromatic information could have implications for the detection and classification of pathological features associated with various eye diseases.

The aim of this work is to evaluate the color rendering of color fundus photographs acquired with the white LED light confocal system and compare it to the rendering from a conventional fundus camera through the use of chromaticity analysis.

## Methods

### Study design

This is a prospective, observational, cross-sectional case series. The study protocol follows the tenets of the Declaration of Helsinki (2013) and was approved by the Istituto Europeo di Microchirurgia Oculare - IEMO review board (approval number 2017.2805A). Written, informed consent was obtained from all the participants before entering the study.

### Study population

Consecutive patients, aged 18 or over, were recruited and enrolled at the Istituto Europeo di Microchirurgia Oculare - IEMO (Udine, Italy) between September 2017 and December 2017. Patients were excluded from the study if they were unable to give informed consent, to be positioned at the slit lamp table, or to fixate on the light target of the camera.

### Study protocol

Each subject underwent a complete ophthalmologic examination, including best-corrected visual acuity (BCVA) assessment on standard Early Treatment Diabetic Retinopathy Study (ETDRS) charts, slit-lamp biomicroscopy, and dilated ophthalmoscopy. On the same day, nonmydriatic fundus images were acquired using a fully automated retinal imaging system (Eidon, Centervue, Padova, Italy) (system 1) and a conventional flash fundus camera (TRC-NW8, Topcon Corporation, Tokyo, Japan) (system 2).

According to the protocol, one retinal image centered on the macula was captured for each eye by a trained technician. Care was taken to generate gradable quality images.

All color images were evaluated with respect to chromaticity. Images were analyzed exactly as they were outputted from the two devices. No image processing (e.g. tone and contrast enhancing/adjustment or color normalization) was performed.

### Fundus cameras

#### System 1

**Fig. 1:**
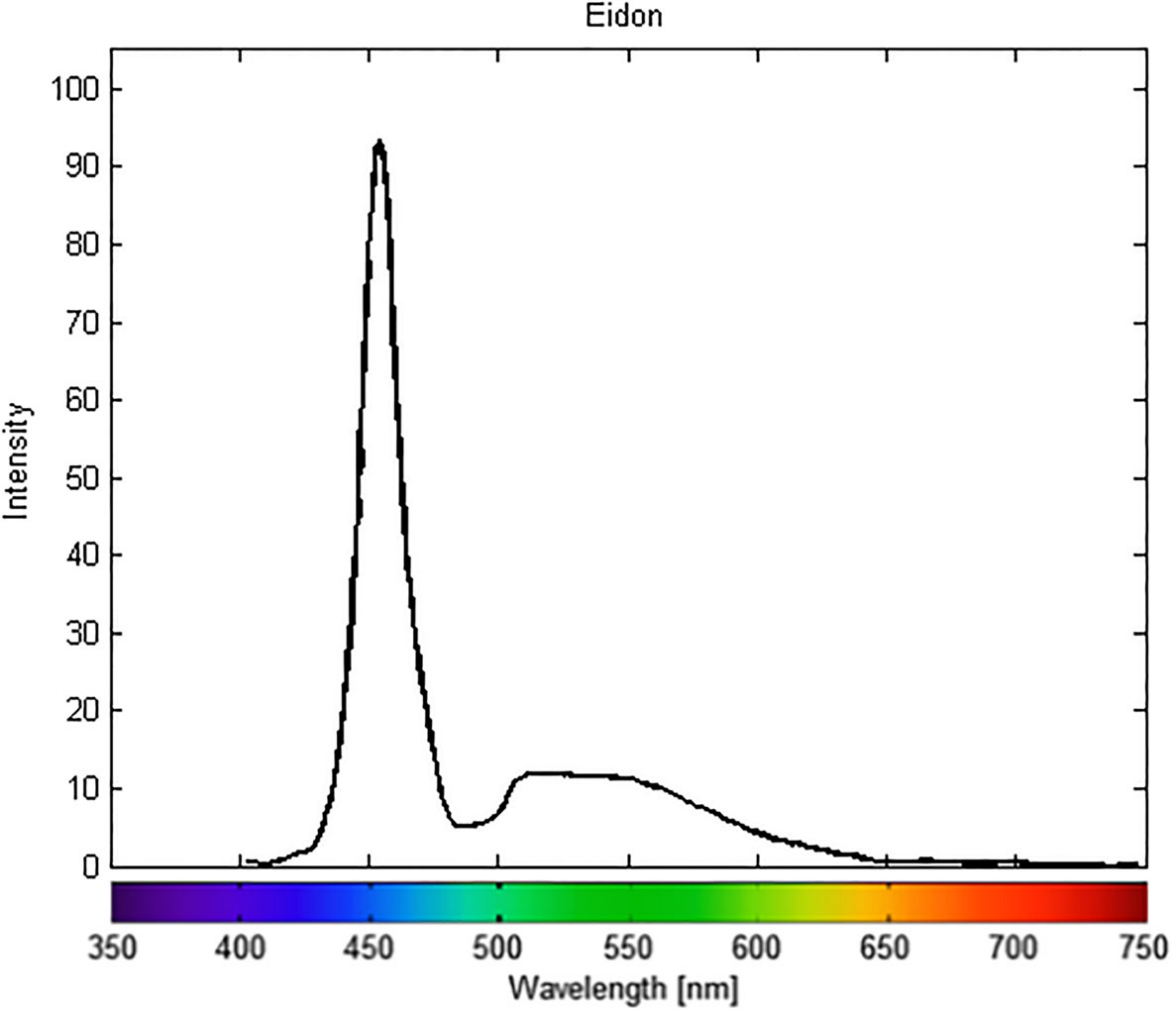
Emission spectrum of the white-light LED illumination of the confocal Eidon system. The spectrum shows peaks at 455 and 500–550 nm with a relatively narrow bandwidth

The Eidon device is a slit confocal system that captures 60-degree, 14-megapixel retinal images in an automated fashion through a nonmydriatic pupil (as small as 2.5 mm). The light source is a broad spectrum white-light LED (440–650 nm). The emission spectrum of the white-light LED is provided in Fig. 1.

#### System 2

**Fig. 2:**
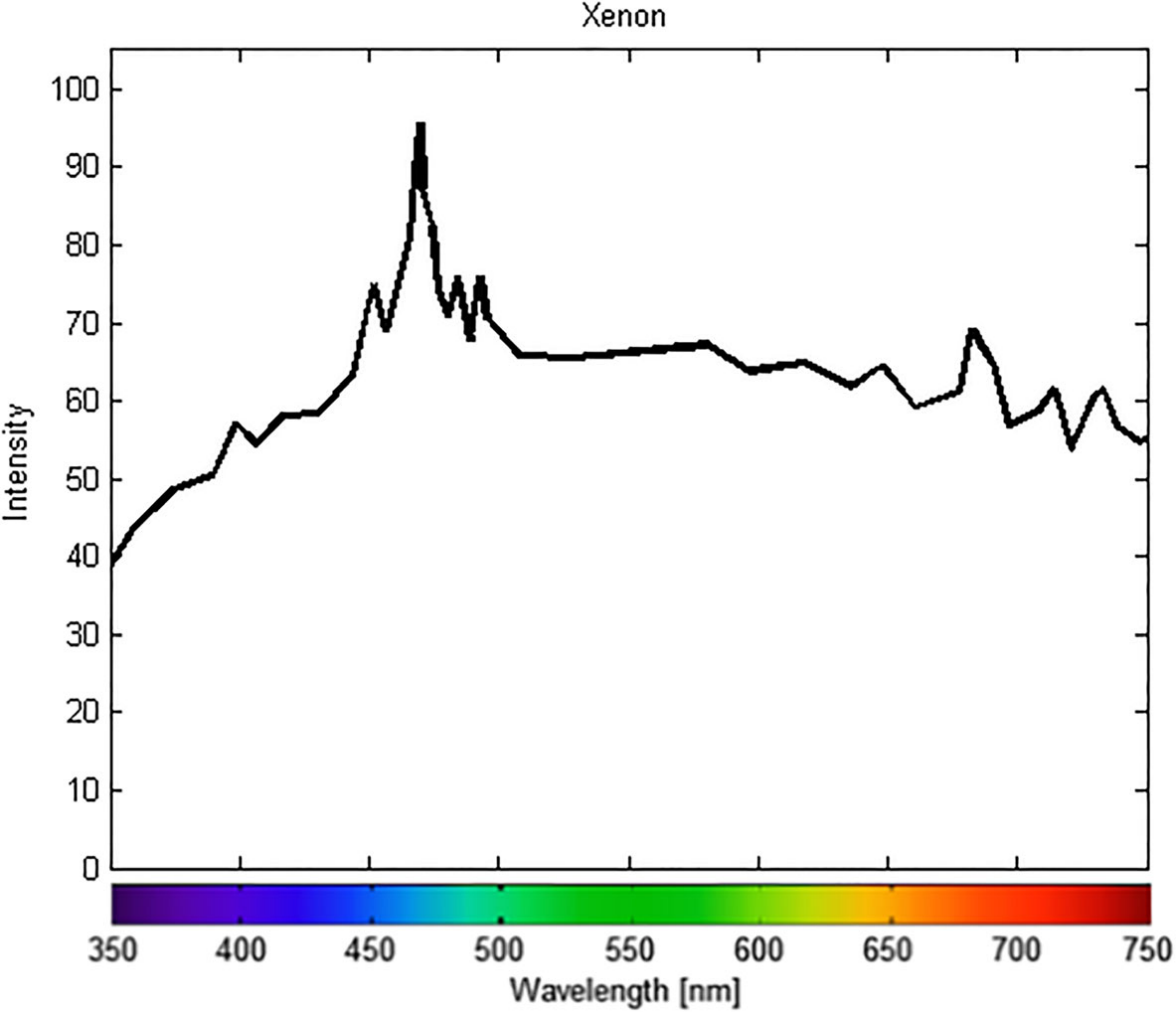
Emission spectrum of the xenon bulb light source of the flash-based fundus camera. The emitted wavelengths show a peak at 480 nm with a wide and continuous bandwidth

A high-definition, nonmydriatic color fundus camera was used to acquire 45-degree, 12-megapixel digital images. The system is capable of capturing images through pupils as small as 3.3 mm in size and features a xenon light source. The xenon lamp emission profile is provided in Fig. 2.

### Chromaticity analysis

**Fig. 3:**
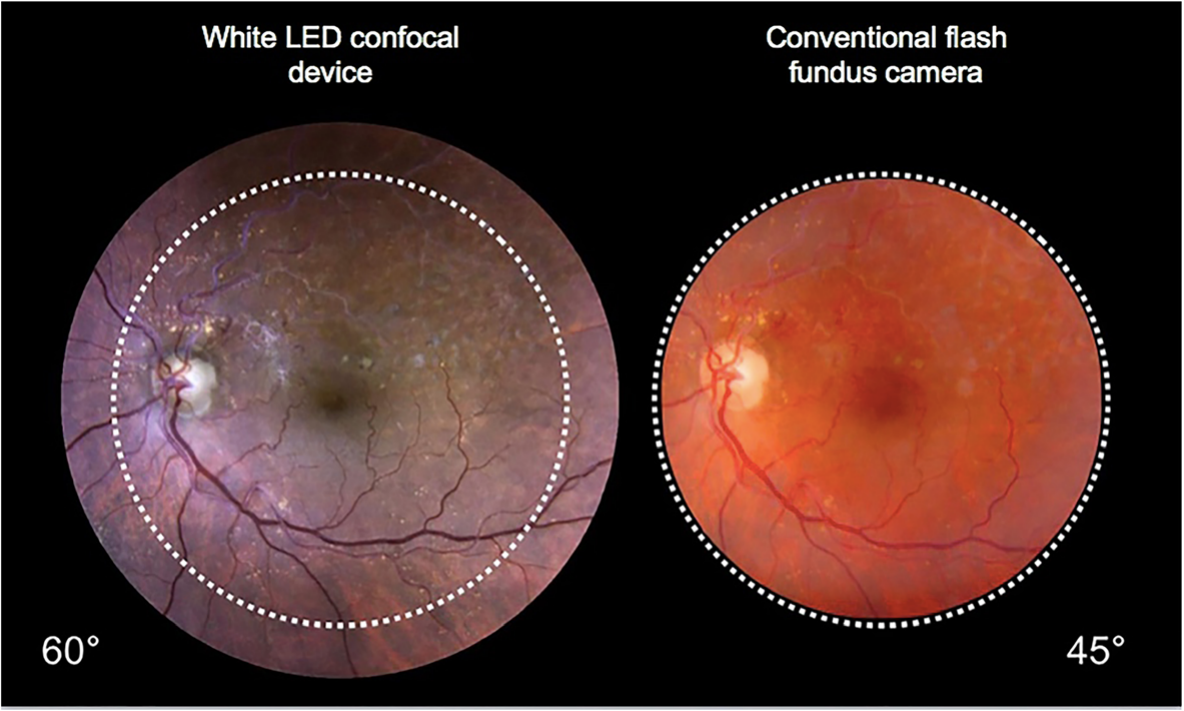
On the left, an image from a case of branch retinal vein occlusion acquired with the confocal LED device. On the right, an image of the same patient captured with the conventional fundus camera. Since both the confocal LED device and the conventional fundus camera produced images with different angles of view, the images were cropped, and chromaticity analysis was performed on the same retinal field size

Since the two devices produce images with different angles of view, for the purpose of performing chromaticity analysis, we cropped the images to have the same retinal field size for evaluation (Fig. 3).

The images taken from the two devices were compared in a structured color space, i.e., a mathematical model where each color can be represented by a set of coordinates [13, 14]. Since the color of a single pixel corresponds to a position in the color space, indicated by its coordinates, the totality of the pixels in an image defines a region that is a subset of the whole color space. This region is distinctive for every image; thus, we termed it the *color signature*.

In this study, we used the default *RGB color space* and the *rgb chromaticity color space* [15]*.* Whereas in the *RGB* (Red, Green, Blue) *color space*, a single pixel is identified by the intensity of red, green, and blue primary colors, the same pixel in the *rgb chromaticity space* model is represented by the normalization of its RGB intensities:

1 $$ {\displaystyle \begin{array}{c}r=\frac{R}{R+G+B}\\ {}g=\frac{G}{R+G+B}\\ {}b=\frac{B}{R+G+B}\end{array}} $$

2 $$ r+g+b=1 $$

Through the rest of this article, we will refer to the primary color intensities with uppercase letters and to their normalized values (the chromaticity) with lowercase letters. Since *rgb chromaticity* is normalized over intensity, its descriptive power is invariant to illumination and related only to the quality of the color. For example, a dark, pure red, represented as *RGB* = [50, 0, 0], is different than a bright, pure red, represented as *RGB* = [200, 0, 0]; in the *chromaticity space*, where a color is represented by the proportions of intensities rather than by the intensities themselves, both the dark and bright pure reds are expressed as *rgb* = [1, 0, 0].

By the definitions given in Eq. 1, the sum of *r, g, and b* will always be equal to one (Eq. 2); because of this property, the *b* dimension can be ignored without causing any loss of information. In fact, if we know the *r* and *g* components of a single pixel, we can always retrieve the *b* component by:

3 $$ b=1-r-g $$

Thus, the *color signature* of an image can be displayed as a distribution of points in the *r and g* axes of the *chromaticity space.* In the *rg chromaticity space,* the horizontal axis represents the *r* component, and the vertical axis represents the *g* component; as stated above, the third coordinate (*b*) can always be inferred (Eq. 3). The origin *rg = [0, 0]* corresponds to pure blue*, rg = [1, 0]* to pure red, and *rg = [0, 1]* to pure green; $$ rgb=\left[\raisebox{1ex}{$1$}\!\left/ \!\raisebox{-1ex}{$3$}\right.,\raisebox{1ex}{$1$}\!\left/ \!\raisebox{-1ex}{$3$}\right.,\raisebox{1ex}{$1$}\!\left/ \!\raisebox{-1ex}{$3$}\right.\right] $$ is the location of all shades of gray (from black to white).

### Outcomes

The color signature of a single image can be synthesized using three parameters derived from the analysis of its pixel distribution on the *rgb chromaticity space*:

- the *barycenter* [*μ*_*r*_, *μ*_*g*_, *μ*_*b*_], computed as the average of the chromaticity of all pixels and representing the center around which the other values are distributed::

4 $$ {\displaystyle \begin{array}{l}{\mu}_r=\frac{1}{N}{\varSigma}_Nr,\kern0.5em {\mu}_g=\frac{1}{N}{\varSigma}_Ng,{\mu}_b=\frac{1}{N}{\varSigma}_Nb\\ {} with\ N\  the\ number\ of\ pixels\ in\ the\ image\end{array}} $$

- The *variability* [*σ*_*r*_, *σ*_*g*_, *σ*_*b*_], computed as the standard deviation of the chromaticity value of all pixels and representing the diversity of colors in the distribution::

5 $$ {\displaystyle \begin{array}{c}{\sigma}_r=\sqrt{\Big[}\frac{1}{N}{\varSigma}_N{\left(r-{\mu}_r\right)}^2\left],{\sigma}_g=\sqrt{\Big[}\frac{1}{N}{\varSigma}_N{\left(g-{\mu}_g\right)}^2\right],{\sigma}_b=\sqrt{\Big[}\frac{1}{N}{\varSigma}_N{\left(b-{\mu}_b\right)}^2\Big]\\ {} with\ N\  the\ number\ of\ pixels\ of\ the\ image\end{array}} $$

- The *number of unique colors* (NUC), which is the total number of different locations covered by the distribution in the *chromaticity space*, represents the quantity of different colors expressed by the image. Since an image’s NUC is an area, it is computed as a percentage of the total area of the *chromaticity space.*

From a diagnostic point of view, a good color image has a high descriptive power when it is characterized by a barycenter close to the center of the chromaticity diagram (i.e., $$ rgb=\left[\raisebox{1ex}{$1$}\!\left/ \!\raisebox{-1ex}{$3$}\right.,\raisebox{1ex}{$1$}\!\left/ \!\raisebox{-1ex}{$3$}\right.,\raisebox{1ex}{$1$}\!\left/ \!\raisebox{-1ex}{$3$}\right.\right] $$) and is surrounded by a wide and continuous cloud of pixels.

To characterize the capability of a device to provide images with good color signatures, a large series of images must be analyzed. We devised a set of descriptors that are computed on a population of image signatures:

- The average of the *barycenter* positions of all the signatures, computed as AVG (*μ*_*r*_), AVG (*μ*_*g*_), and AVG (*μ*_*b*_)
- The variability (standard deviation) of the *barycenter* positions of all the signatures, computed as SD (*μ*_*r*_), SD (*μ*_*g*_), and SD (*μ*_*b*_)
- The average of the *variability* (standard deviation) of all the signatures, computed as AVG (*σ*_*r*_), AVG (*σ*_*g*_) and AVG (*σ*_*b*_)
- The average *NUC* of all the signatures

Thereafter, a good color imaging device, developed specifically for the diagnosis of retinal pathologies, is identified by an average *barycenter* located close to the center of the color space (no color dominance), a high variability of the barycenter position (different retinal conditions are represented with different color signatures), a high standard deviation (the retina is reproduced with a collection of distant colors), and a high *NUC* percentage (the device is able to express a continuum of different colors) [16–20].

### Statistical analysis

After assessing the normality of the distributions (Shapiro-Wilk test), differences in chromaticity analysis outcomes and in positions in the color space were evaluated using a two-tailed paired t-test and multivariate paired Hotelling’s T^2^. A *p* value of <0.05 was defined as statistically significant.

## Results

**Fig. 4:**
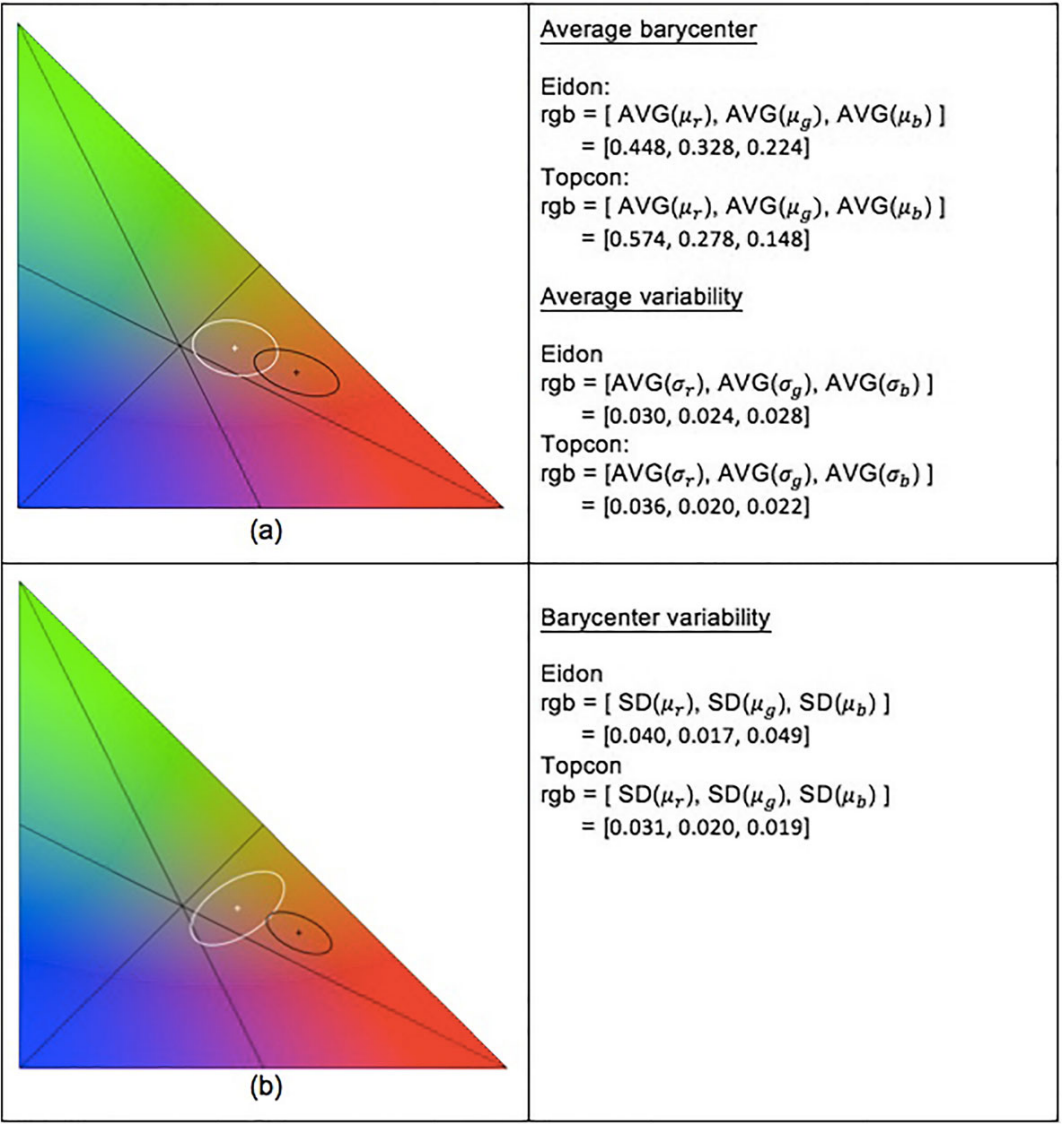
**a** The crosses are the average (AVG) position of the chromaticity barycenters for the confocal LED (white) and flash color fundus (black) devices; the ellipses represent bivariate Gaussian distributions with standard deviations (SD) equal to the average variability of chromaticity computed on the population of signatures; ellipse area comprises 95% of the distribution. **b** Ellipses here represent bivariate Gaussian distributions with standard deviations equal to the barycenter variability computed on the population of barycenters; ellipse area comprises 95% of the distribution

Confocal white LED color and flash color fundus images were obtained from 233 eyes of 181 patients. The patient characteristics are detailed in Table 1. A wide variety of diseases were included, although over one third of the eyes showed evidence of age-related macular degeneration (AMD). A statistically significant difference in the average *barycenter* position in the chromaticity space was recorded between the two color-imaging devices (*p* < 0.001) (Fig. 4).

**Table 1:** Baseline characteristics

Eyes (n)	233
Age (mean ± SD)	67.4 ± 9.2
Female (%)	35
Normal fundus (n)	40
Retinal diseases (n)	193
AMD	88
Retinal dystrophies	25
DR	23
PM	13
VMI diseases	12
RVO	11
Central serous chorioretinopathy	6
Ocular tumors	4
RAO	4
Other Vascular Diseases	4
Retinal detachment	3

Legend: *AMD* Age-related macular degeneration, *DR* Diabetic retinopathy, *PM* Pathologic myopia, *n* Number, *RAO* Retinal artery occlusion, *RVO* Retinal vein occlusion, *SD* Standard deviation, *VMI* Vitreomacular interface

The images acquired by system 1 demonstrated an average *barycenter* position (rgb = [0.448, 0.328, 0.224]) closer to the center of the chromaticity space ($$ rgb=\left[\raisebox{1ex}{$1$}\!\left/ \!\raisebox{-1ex}{$3$}\right.,\raisebox{1ex}{$1$}\!\left/ \!\raisebox{-1ex}{$3$}\right.,\raisebox{1ex}{$1$}\!\left/ \!\raisebox{-1ex}{$3$}\right.\right] $$), while system 2 resulted in images with a clear shift toward red at the expense of blue and green (rgb = [0.574, 0.278, 0.148]) (*p* < 0.001).

The variability of the barycenter positions was higher in the system 1 camera than in the system 2 camera. Furthermore, the standard deviation values for the *r*, *g* and *b* chromaticities were 0.040, 0.017 and 0.049, respectively, using the confocal white-light imaging system and 0.031, 0.020 and 0.019, respectively, using the conventional flash fundus camera.

The average variability of the distributions was higher (0.003 ± 0.007, *p* < 0.001) in system 1 images compared to system 2 images. Specifically, the values for the *r*, *g* and *b* axes were 0.036, 0.024 and 0.028, respectively, with the confocal white-light device and 0.036, 0.020 and 0.022, respectively, using the flash camera. The NUC percentage was higher for system 1 than for system 2 (0.071% versus 0.025%, *p* < 0.001).

The results of the chromaticity analysis are summarized in Table 2.

**Table 2:** Results of chromaticity analysis

Chromaticity signature descriptor	Color Channel	Eidon	Topcon camera	Relative change Eidon vs Topcon camera (%)
Average barycenter (AVG)	r	0.448	0.574	−21.95
	g	0.328	0.278	18.28
	b	0.224	0.148	50.67
Average variability (AVG of SD)	r	0.036	0.036	0.55
	g	0.024	0.020	19.02
	b	0.028	0.022	28.09
Barycenter variability (SD)	r	0.040	0.031	28.55
	g	0.017	0.020	−16.57
	b	0.049	0.019	164.70
Average NUC (AVG)	%	0.071	0.025	180.96

Legend: *b* Blue, *g* Green, *r* Red, *AVG* Average, *SD* Standard deviation

**Fig. 5:**
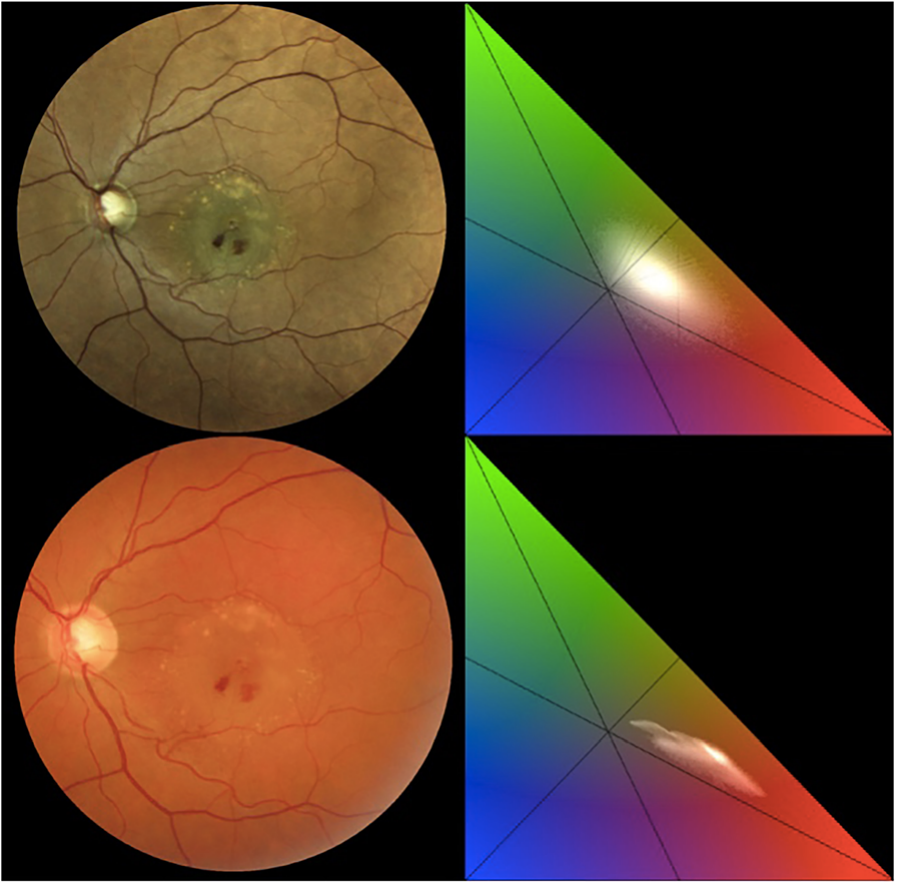
On the top row, the image and the chromaticity analysis of a case of retinal angiomatous proliferation (RAP) acquired with the confocal LED device (system 1). On the bottom row, the image and the chromaticity analysis of the same patient captured with conventional fundus camera (system 2). The color analysis of the retinal image, performed with both devices, shows that system 1 provides an image with a barycenter position closer to the center of the color space compared to the same image acquired with system 2, whose barycenter is notably moved toward the red channel. Comparing the two images, system 2 provides a color fundus picture that appears flat and reddish

**Fig. 6:**
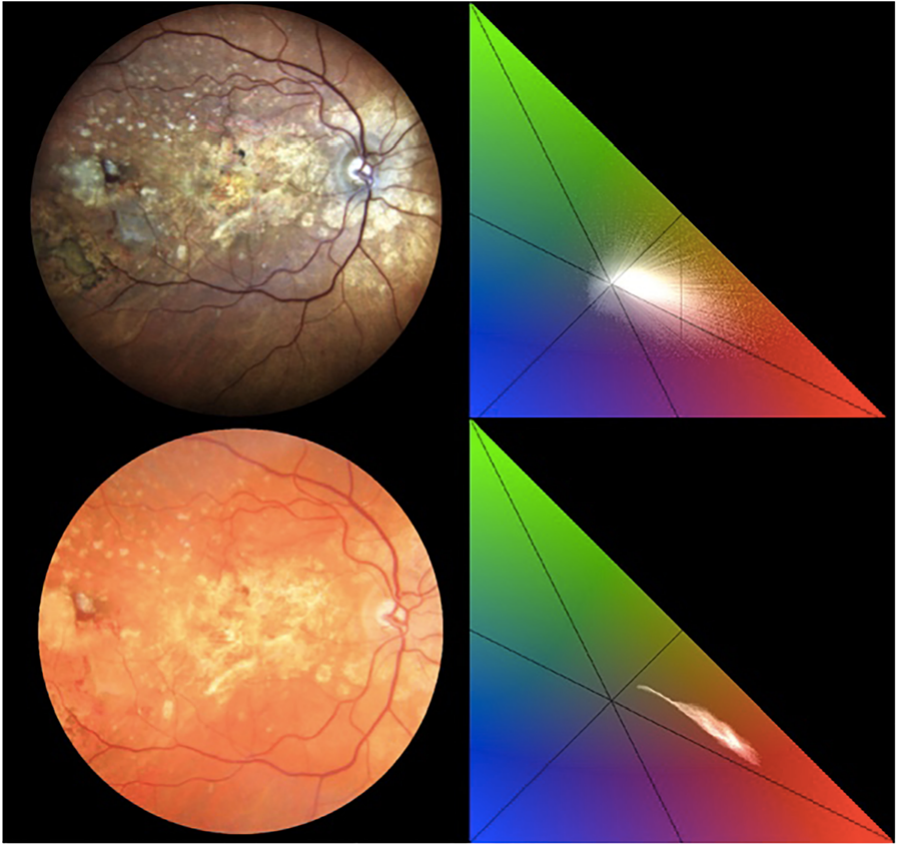
On the top row, an image and the chromaticity analysis of a case of exudative age-related macular degeneration acquired with the confocal LED device (system 1). On the bottom row, an image and the chromaticity analysis of the same patient captured with the conventional fundus camera (system 2). The chromaticity evaluation of the retinal image acquired with system 1 shows that the cluster of pixels is wider in comparison with the same image captured with system 2. Overall, a richer color content is evident in system 1

Two clinical examples are shown in Fig. 5 and Fig. 6.

## Discussion

Diagnostic accuracy by means of photographic documentation is highly dependent on the quality of images because poor image quality can impair the visualization of characteristic disease features. The color characteristics are key in distinguishing different features, such as hemorrhage, pigments, or lipids, which may sometimes have overlapping morphologic characteristics. Thus, accurate color rendering may be vital.

The *RGB color space* and the *rgb chromaticity space* are useful abstract mathematical models for evaluating the capabilities of a digital camera to describe, classify and compare color attributes of an acquired image. A mathematical analysis of the chromaticity can also show whether different devices are able to faithfully render colors and highlight details, giving an indirect evaluation of the relative abilities to discern pathologic signs [15].

The results of our study show that the Eidon confocal, white LED fundus camera system provides a well-balanced color image because the barycenter position is generally located very close to the center of the *rgb chromaticity* space. By contrast, pictures acquired by the conventional flash-based fundus camera are characterized by a high predominance of red, resulting in an oversaturated and potentially less informative retinal image.

The richness of the color content of an image is quantified by a measure of the dispersion of the pixels around its barycenter. Chromaticity analysis of our study sample showed that images acquired with Eidon offer a wider cluster of pixels in comparison with the same image captured with the conventional fundus camera. This suggests that there is a broader range or greater “richness” of color in the confocal LED image.

Another key aspect for assessing the color capability of an imaging device is the size of the color gamut, the range of color that a device is able to display in relation to the RGB color space. The normalized chromaticity value is a measure that defines the color gamut of a specific digital camera. In our study, the normalized chromaticity value is higher for the confocal LED system than for the conventional fundus camera. This finding highlights that a conventional system provides images with a smaller color gamut in the RGB space, which means that it covers a smaller range of colors. Hence, the confocal color system is able to provide images with a wider representation of colors. This may theoretically provide greater contrast for disease feature assessment. Contrast, or the ability to distinguish a pathological expression from a normal background, is essential to the diagnostic accuracy of a system. In color retinal imaging, this attribute is based on chromaticity discrimination, which refers to the ability of an observer to distinguish two colors. It can be measured as the minimum variation in chromaticity needed to achieve a minimally noticeable difference from any point in the color space diagram. This analysis results in a MacAdam ellipse, which is a region on a chromaticity diagram that contains all colors that are indistinguishable, to the average human eye, from the color at the center of the ellipse [21, 22].

From MacAdam’s studies, two main conclusions can be drawn. First, given two image signatures with the same dispersions, human vision is more sensitive to differences in the one that ranges through different colors than in the one that lies on the same tonality (e.g., a barycenter located at the center of the gamut triangle vs. at the green and red periphery). Second, the wider the cluster of pixels surrounding the barycenter, the higher the probability that two points of interest are located far from each other on the chromaticity diagram. Conversely, when the cluster of pixels is concentrated around the barycenter, it is more probable that the two points are located within the same MacAdam ellipse and thus are indistinguishable [21].

**Fig. 7:**
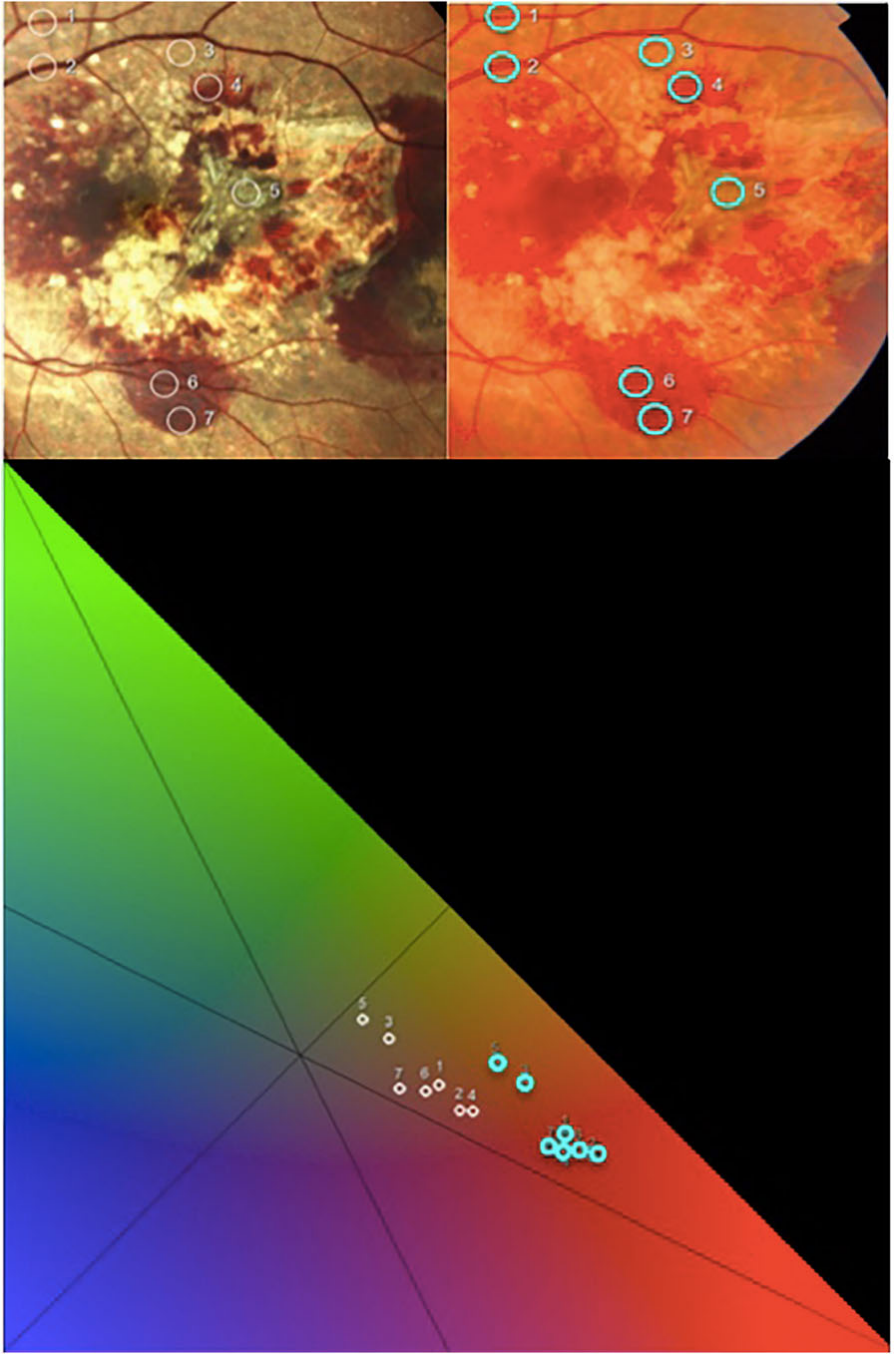
Seven reddish points of the same retinal field captured with the confocal LED device (white rings) and with the conventional camera (light blue rings) plotted on the chromaticity diagram

To further clarify these concepts, we plotted seven reddish points of the same retinal field captured with the confocal LED system and with the flash color fundus camera in the chromaticity diagram. It can be easily appreciated that the reds acquired with the confocal LED system are set at a greater distance than those acquired with the flash color device (Fig. 7). This reflects the fact that in this case, the confocal system is able to make the color discrimination more noticeable for human eyes.

A final point that merits discussion is the wider variability of the barycenter position in images acquired with the confocal LED device. This finding mirrors an important characteristic of photographic instruments: when images of different fundi are captured, a device able to produce signatures with different barycenters should be considered more capable and versatile than one that always generates the same barycenter.

We may infer that the confocal LED system produces color images with a greater descriptive power compared with a traditional flash-based fundus camera because it produces color images with a greater richness of color content.

There are technical reasons that account for these observed differences in color rendering between the two instruments. First, the two devices use different light sources. The confocal device utilizes a white-light LED illumination, while the flash color camera uses a xenon bulb as the light source. It is well known that colors look different depending on the spectral characteristics of the light source (Fig. 1 and Fig. 2). Moreover, the different on-device image processing algorithms employed in the two fundus cameras can either emphasize red and orange wavelengths over blues and greens (system 2) or produce a smoother color curve, with appropriate representations of the blue and green wavelengths (system 1). A second and critical difference is the confocal attribute of the LED system. By using a slit confocal aperture, light reflected from out-of-focus layers is masked and provides only a marginal contribution to image formation. This typically reduces the choroidal contribution to the red channel, which may otherwise “fog” or reduce contrast in the image.

A critical aspect of Eidon is that this device is set up to produce a greater blue component than conventional flash-based fundus cameras. On the on one hand, this lends the image a broader representation of colors, which improves the quality of the image itself and yields a clearer visualization of retinal details; on the other hand, this image does not correspond to what ophthalmologists are used to seeing during routine clinical examinations. Similar to traditional flash-based fundus cameras, the fundus oculi looks very reddish using an ophthalmoscope or during a slit-lamp biomicroscopy. This is due to the redshifted emission spectrum of the light source used by indirect ophthalmoscopes and slit lamps and to the visualization of the blood-filled choroid that cannot be isolated from the overlying neuroretinal tissue by the human eye during clinical examination.

## Conclusions

In conclusion, in this study we demonstrated that the new confocal, white-light LED system, Eidon, produces color images which are more balanced (i.e.,. not saturated by the red component) and “richer” (greater color discrimination and broader gamut) compared with those obtained with a traditional flash color fundus camera. These benefits should theoretically yield greater discriminative power and diagnostic accuracy, providing an accurate documentation of retinal appearance. We believe that this new fundus imaging system has the potential to enhance patient care, especially when employed together with artificial intelligence-based algorithms in screening programs. This is particularly true for conditions like diabetic retinopathy, where most of the retinal findings are reddish (e.g., new. Epiretinal vessels, intraretinal microvascular abnormalities, microaneurysms, intraretinal hemorrhages) and can be more easily distinguished from the background when using a device with a broader gamut. To draw a definitive conclusion on the clinical utility of this new device, a prospective study comparing the performance of human graders and artificial intelligence-based algorithms when using either a conventional fundus camera or Eidon is warranted.

## Data Availability

The datasets used and/or analyzed during the current study are available from the corresponding author upon reasonable request.

## Abbreviations

AMD: Age-related macular degeneration
AVG: Average
BCVA: Best-corrected visual acuity
cSLO: Scanning laser ophthalmoscopy
DR: Diabetic retinopathy
LED: Light Emitting Diode
n: Number
NUC: Number of unique colors
PM: Pathologic myopia
RAO: Retinal artery occlusion
RAP: Retinal angiomatous proliferation
RGB: Red-green-blue
RVO: Retinal vein occlusion
SD: Standard deviation
VMI: Vitreomacular interface
